# The complete chloroplast genome of *Araucaria cunninghamii* (Araucariaceae)

**DOI:** 10.1080/23802359.2020.1787265

**Published:** 2020-07-25

**Authors:** Jingyao Ping, Xin Luo, Ming Zhu, Rongjing Zhang, Chunmei Qian, Yingjuan Su, Ting Wang

**Affiliations:** a College of Life Sciences, South China Agricultural University, Guangzhou, China; b Institute of Tropical and Subtropical Cash Crops, Yunnan Academy of Agricultural Sciences, Baoshan, China; c School of Life Sciences, Sun Yat-sen University, Guangzhou, China; d Research Institute of Sun Yat-sen University in Shenzhen, Shenzhen, China; e Guangdong Provincial Key Laboratory of Applied Botany, South China Botanical Garden, Chinese Academy of Sciences, Guangzhou, China

**Keywords:** *Araucaria cunninghamii*, chloroplast genome, phylogenetic analysis

## Abstract

The chloroplast genome of the *Araucaria cunninghamii* has been completely sequenced. The genome size is 146,337 bp, and the overall GC content is 36.7%. This cp genome does not contain cannonical IRs, it encodes a total 122 genes including 82 protein-coding genes, 36 tRNA genes and four rRNA genes. Among them, eight protein-coding genes (*rpl*2, *rpl*16, *pet*B, *pet*D, *rpo*C1, *atp*F, *ndh*A and *ndh*B) have two exon, and two genes (*rps*12 and *ycf*3) have three exons. Also, *trn*I-CAU and *rrn*5 has two copies. The Maximum-likelihood tree shows that *A. cunninghamii* is sister to *A. heterophylla*.

*Araucaria cunninghamii* (Araucariaceae) is a perennial evergreen tree. It is native to the southeastern coastal area of Oceania and is the main afforestation tree species in tropical and subtropical regions. Its tall tree shape and graceful posture are known as one of the five major park tree species in the world and are important ornamental plants in the garden, and cultivated in many places in China (Zheng and Fu 1978). *A*. *cunninghamii* is a common group of *Araucaria*, and its chloroplast genome characteristics have not been published. Obtaining its complete chloroplast genome can provide molecular resources for the study of phylogenetic genomics and population variation of *A. cunninghamii*.

Fresh leaves were sampled from the campus of South China Agricultural University (SCAU), location (E113°20′, N23°9′). The specimens were stored in Herbarium of the College of Life Sciences, SCAU (specimen no.: PJY-NYS1910). Genomic DNA was extracted using Tiangen Plant Genomic DNA Kit (Tiangen Biotech Co., Beijing, China). Illumina HiSeq 2500 platform was used for sequencing. A total of 12,206,029 raw reads were generated. Clean reads were obtained after filtering with Trimmomatic v0.32 (Bolger et al. 2014), which were *de novo* assembled into contigs by Velvet (Zerbino and Birney 2008). DOGMA (Wyman et al. 2004) was used for gene prediction. The protein sequence obtained by gene prediction was compared with the NCBI CDD (Marchler-Bauer et al. 2015, Conserved Domain Database) database, and COG function information of genes was predicted by rpsblast v2.2.30+ (Altschul et al. 1997). The ClustalW model in MEGA X (Kumar et al. 2018) was used to create a multiple sequence alignment of the complete cp genome of *A. cunninghamii* with those of other 12 plants downloaded from GenBank. Based on maxium-likelihood (ML), the phylogenetic tree was constructed using RaxMLGUI2 (Stamatakis 2014) with 1000 bootstrap replicates.

The complete cp genome of *A. cunninghamii* is 146,337 bp in size with circular DNA molecular structure (GenBank accession number: MT227812). Like other conifers, it does not contain inverted repeat (IR) region. The cpDNA contains 122 genes, including 82 protein-coding genes, 36 tRNA genes and four rRNA genes. Among these genes, 14 genes (eight protein-coding genes and six tRNA genes) contain one introns, two genes (*rps12*, *ycf3*) have two introns. Like other species of *Araucaria* (such as *A*. *angustifolia*) (Brandão et al. 2019), it also has two duplicated genes (*trn*I-CAU and *rrn*5). The ML-tree highly supports *A. Cunninghamii* and *A. heterophylla* are sister groups, and the monophyletic clade formed by them are sister groups of remaining *Araucaria* (Figure 1). The cp genome of *A. cunninghamii* provides a reliable molecular resource for phylogenetic studies and chloroplast genomics of gymnosperms.

**Figure 1.**
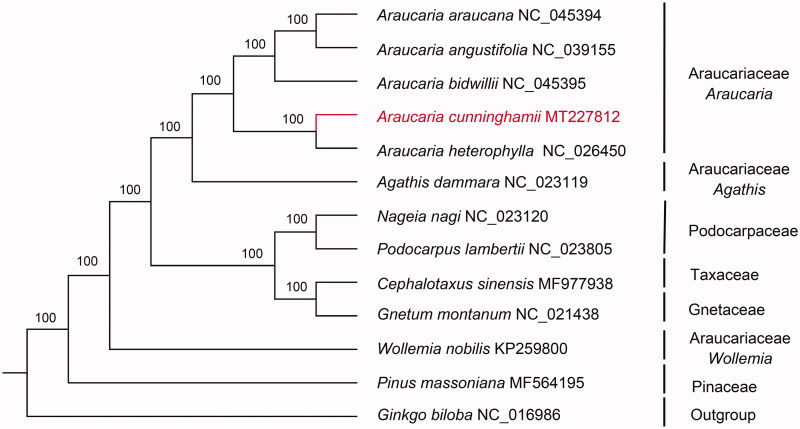
The ML phylogenetic tree constructed based on the complete chloroplast genome sequences of 13 species. Outgroup is *Ginkgo biloba*. Bootstrap support values are marked on nodes.

## Data Availability

The data that support the findings of this study are available in [NCBI] at [https://www.ncbi.nlm.nih.gov/]. These data were derived from the following resources available in the public domain: [https://www.ncbi.nlm.nih.gov/nuccore/NC_021438,KP259800,MF977938,MT227812,NC_039155,NC_045394,NC_045395,NC_026450,NC_023805,MF564195,NC_023120,NC_016986]
